# Deca­chloro­cyclo­penta­silanes coordinated by pairs of chloride anions, with different cations, but the same solvent mol­ecules

**DOI:** 10.1107/S2056989017016310

**Published:** 2017-11-21

**Authors:** Maximilian Moxter, Julian Teichmann, Hans-Wolfram Lerner, Michael Bolte, Matthias Wagner

**Affiliations:** a Institut für Anorganische Chemie, J. W. Goethe-Universität Frankfurt, Max-von-Laue-Strasse 7, 60438 Frankfurt/Main, Germany

**Keywords:** crystal structure, deca­chloro­cyclo­penta­silanes, inverse-sandwich complex, C—H⋯Cl contacts

## Abstract

We have determined the crystal structures of two deca­chloro­cyclo­penta­silanes, namely bis­(tetra-*n*-butyl­ammonium) dichloride deca­chloro­cyclo­penta­silane di­chloro­methane disolvate, 2C_16_H_36_N^+^·2Cl^−^·Si_5_Cl_10_·2CH_2_Cl_2_, (I), and bis­(tetra­ethyl­ammonium) dichloride deca­chloro­cyclo­penta­silane di­chloro­methane disolvate, 2C_8_H_20_N^+^·2Cl^−^·Si_5_Cl_10_·2CH_2_Cl_2_, (II), both of which crystallize with discrete cations, anions, and solvent mol­ecules. In (I), the complete deca­chloro­cyclo­penta­silane ring is generated by a crystallographic twofold rotation axis. In (II), one cation is located on a general position and the other two are disordered about centres of inversion. These are the first structures featuring the structural motif of a five-membered cyclo­penta­silane ring coordinated from both sides by a chloride ion. The extended structures of (I) and (II) feature numerous C—H⋯Cl inter­actions. In (II), the N atoms are located on centres of inversion and as a result, the ethyl­ene chains are disordered over equally occupied orientations.

## Abstract 2

The planar deca­chloro­cyclo­penta­silane rings in the title compounds are coordinated by two chloride ions to generate inverse-sandwich complexes.

## Chemical context   

The title compounds are the first known halide diadducts of the long-known perchlorinated cyclo­penta­silane Si_5_Cl_10_ (Hengge & Kovar, 1977 ▸). Their structures can be seen as inverse-sandwich complexes, in which two chloride ions lie above and below the planar five-membered silicon ring.
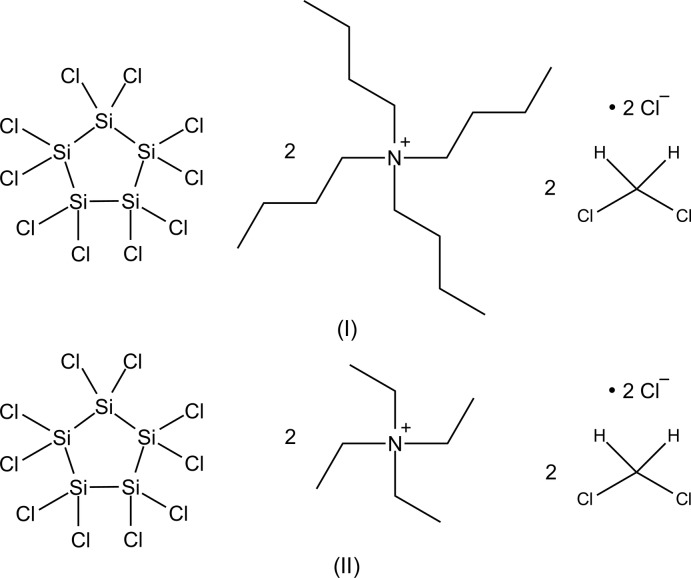


In the recent years, new and facile synthetic protocols for the Cl^−^ diadduct [Si_6_Cl_14_]^2–^ of dodeca­chloro­cyclo­hexa­silane have been developed. It can either be prepared through the chloride-induced disproportionation of Si_2_Cl_6_, which leads directly to [Si_6_Cl_14_]^2–^ (Tillmann *et al.*, 2012 ▸, 2014 ▸; Tillmann, Moxter *et al.*, 2015 ▸), or the Lewis acidic uncomplexed Si_6_Cl_12_ can be used as the starting material. In the latter case, mere addition of soluble chloride salts [*R*_4_N]Cl (*R* = *n*Bu or Et) leads to the formation of [Si_6_Cl_14_]^2–^ (Dai *et al.*, 2010 ▸). Given this background, it was of inter­est to investigate the Lewis acidity and ability of Si_5_Cl_10_ to bind Cl^−^ ions.

## Structural commentary   

**Figure 1:**
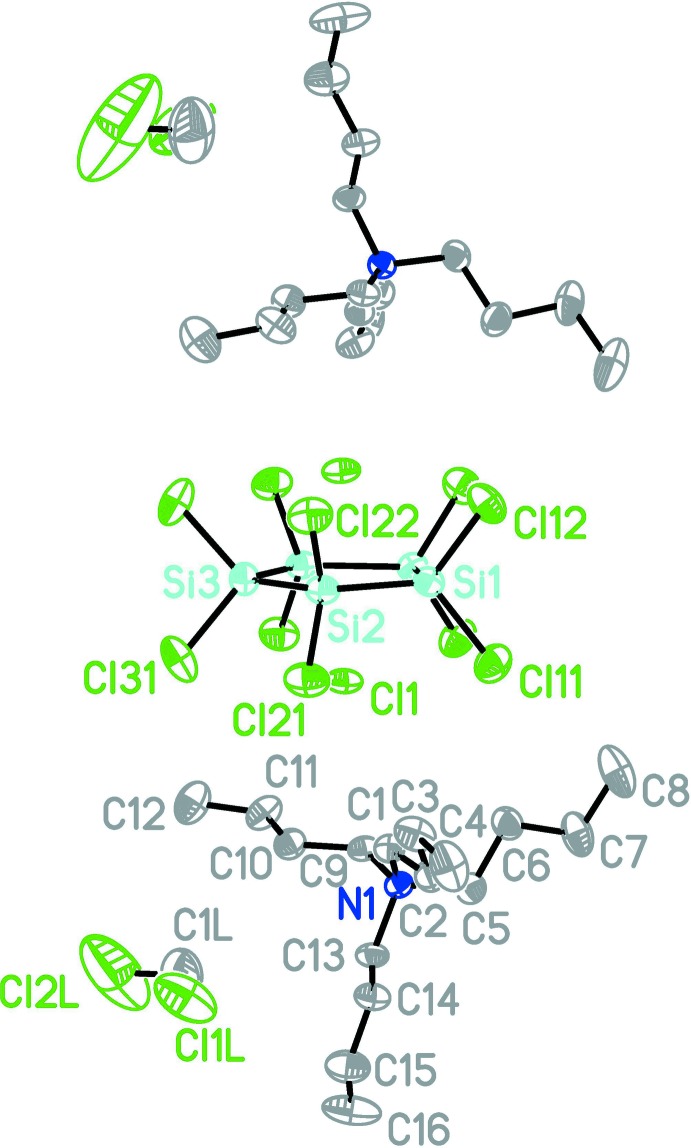
Perspective view of (I) with displacement ellipsoids drawn at the 50% probability level. For clarity, H atoms are omitted and only the symmetry independent mol­ecules are labelled. Atoms without labels are generated by the symmetry operator −*x* + 1, *y*, −*z* + 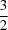.

Bis(tetra-*n*-butyl­ammonium) dichloride deca­chloro­cyclo­penta­silane di­chloro­methane disolvate, 2C_16_H_36_N^+^·2Cl^−^·Si_5_Cl_10_·2CH_2_Cl_2_, (I), crystallizes with discrete cations, anions, and solvent mol­ecules (Fig. 1 ▸). The five-membered deca­chloro­cyclo­penta­silane ring is located on a twofold rotation axis. The Si—Cl bond lengths range from 2.081 (3) Å for Si2—Cl21 to 2.100 (3) Å for Si2—Cl22. The Si—Si bond lengths do not vary markedly: they range from 2.339 (3) Å (Si1—Si2) to 2.347 (3) Å (Si2—Si3).

The almost planar ring (r.m.s. deviation 0.002 Å) is coord­inated by two chloride anions located above and below the ring. The distances of the chloride ions to the Si atoms [Cl1⋯Si1 2.907 (3), Cl1⋯Si2 2.914 (3), Cl1⋯Si3 2.930 (3) Å,] show that the chloride ions are located almost exactly above the centroid of the ring [distance Cl1⋯*Cg* = 2.1434 (16) Å].

**Figure 2:**
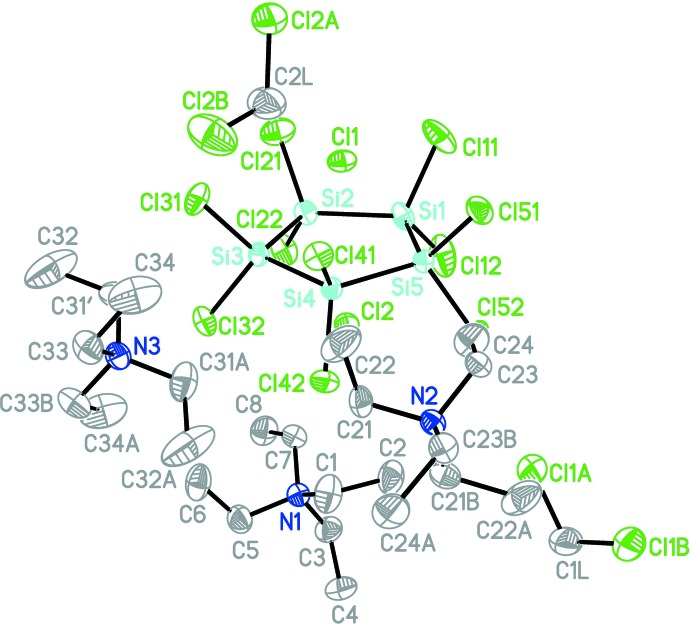
Perspective view of (II) with displacement ellipsoids drawn at the 50% probability level. For clarity, H atoms are omitted and only one of the two disordered sites of the tetra­ethyl­ammonium cations are shown.

Bis(tetra­ethyl­ammonium) dichloride deca­chloro­cyclo­penta­silane di­chloro­methane disolvate, 2C_8_H_20_N^+^·2Cl^−^·Si_5_Cl_10_·2CH_2_Cl_2_, (II), crystallizes as (I) with discrete cations, anions, and solvent mol­ecules (Fig. 2 ▸). The Si—Cl bonds again lie in a quite narrow range [2.0805 (9) Å (Si1—Cl12) to 2.1102 (8) Å (Si2—Cl22)] and the Si—Si bond lengths are also very similar [2.3386 (8) Å (Si1—Si2) to 2.3473 (7) Å (Si4—Si5)].

The five-membered deca­chloro­cyclo­penta­silane ring is almost planar (r.m.s. deviation = 0.017 Å) and coordinated by two chloride anions located above and below the ring with a Cl⋯*Cg* distance of 2.1781 (5) Å for Cl1 and 2.1237 (5) Å for Cl2. The Cl⋯Si distances range from 2.9381 (7) Å (Cl1⋯Si5) to 2.9645 (7) Å (Cl1⋯Si2) and from 2.8759 (8) Å (Cl2⋯Si2) to 2.9510 (7) Å (Cl2⋯Si5). Since the Cl⋯Si distances have a broader range for Cl2, it can be said that this ion is slightly displaced from a position directly over the ring centroid.

It is inter­esting to note that (I) and (II) have – apart from the different cations – the same mol­ecular stoichiometry, *i.e*. one Si_5_Cl_10_ ring coordinated by two chloride anions, two cations and two solvent di­chloro­methane mol­ecules. However, since (I) has twofold rotation symmetry, there are only half of the chemical entities in the asymmetric unit.

## Supra­molecular features   

**Figure 3:**
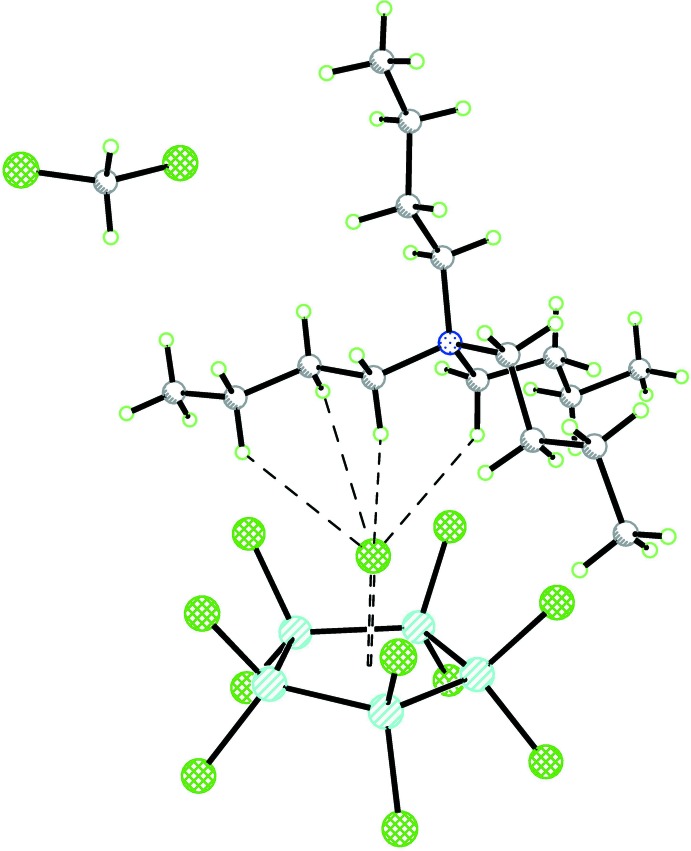
Perspective view of (I) showing the environment of the Cl anion. The contact to the centre of the five-membered ring is drawn as an open dashed bond. H⋯Cl contacts less than 3.5 Å are drawn as dashed lines.

The components of (I) and (II) are linked by a plethora of C—H⋯Cl contacts (Tables 1 ▸ and 2 ▸, respectively); in particular the chloride ions are surrounded by C—H groups. For an example, see Fig. 3 ▸. As a result of the disorder of the N2 and N3 cations in (II), a plot showing the coordination of the Cl ions looks extremely crowded and is therefore omitted.

## Database survey   

The present structures are the first examples of a deca­chloro­cyclo­penta­silane ring coordinated by two anions. There are only two structures of a deca­chloro­cyclo­penta­silane ring in the CSD (Version 5.38 of November 2016 plus three updates; Groom *et al.*, 2016 ▸), namely deca­chloro­cyclo­penta­silane 4-methyl­benzo­nitrile solvate (refcode ELAFON; Dai *et al.*, 2010 ▸) and deca­chloro­cyclo­penta­silane aceto­nitrile solvate (ELAFIH; Dai *et al.*, 2010 ▸). In both of them, the deca­chloro­cyclo­penta­silane ring is almost planar (0.017 Å for ELAFON and 0.001 Å for ELAFIH) and shows almost no variation in the Si—Si (2.358–2.368 Å for ELAFON and 2.342–2.349 Å for ELAFIH) and Si—Cl (2.030–2.059 Å for ELAFON and 2.034– 2.038 Å for ELAFIH) bond lengths.

The distance of the N atom to the centroid of the ring is 2.152 and 2.196 Å for ELAFON and 2.234 Å for ELAFIH. This difference could be due to the steric demand of the benzene ring in ELAFIH. The N⋯*Cg* distances are in the same range as the Cl⋯*Cg* distances in (I) and (II).

Mean values of the structural parameters of the four compared structures and dichloride dodeca­chloro­cyclo­hexa­silanes (Tillmann, Lerner & Bolte, 2015 ▸) are compiled in Table 3 ▸. It is remarkable that the Si—Si and Si—Cl bond lengths do not vary significantly between the five and six-membered Si rings, but the Cl⋯*Cg* distance in the dodeca­chloro­cyclo­hexa­silanes is significantly shorter than for deca­chloro­cyclo­penta­silane. This might be due to the fact that the Cl ligands form a narrower cone in five- compared to six-membered rings.

## Synthesis and crystallization   

**Figure 4:**
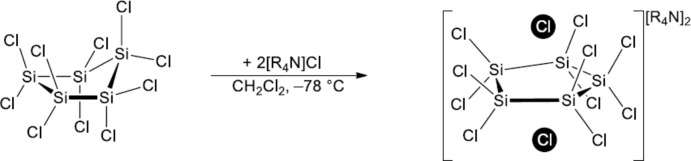
Synthesis of [*R*_4_N]_2_[Si_5_Cl_12_] (*R* = *n*Bu or Et).

**Figure 5:**
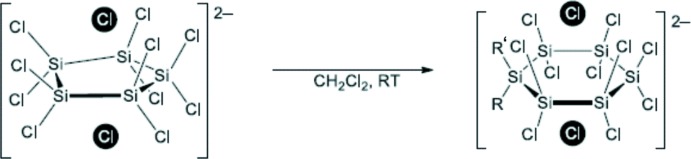
Transformation of [*n*Bu_4_N]_2_[Si_5_Cl_12_] (I) to [*n*Bu_4_N]_2_[Si_6_Cl_14_] (*R* = *R*′ = Cl) and [*n*Bu_4_N]_2_[Si_7_Cl_16_] (*R* = Cl; *R*′ = SiCl_3_).

The addition of a solution of [*R*_4_N]Cl (*R* = *n*Bu or Et) in CH_2_Cl_2_ at 195 K to a solution of Si_5_Cl_10_ in CH_2_Cl_2_ furnished the Cl^−^ diadducts [*R*_4_N]_2_[Si_5_Cl_12_] (*R* = *n*Bu or Et) (Fig. 4 ▸). Crystals of [*R*_4_N]_2_[Si_5_Cl_12_] (*R* = *n*Bu or Et) could be harvested after storage of the reaction solution for one week at 195 K in 89% and 93% yield, respectively. Both adducts are stable in the solid phase under inert conditions. However, in solution a rapid transformation of [*n*Bu_4_N]_2_[Si_5_Cl_12_] to [*n*Bu_4_N]_2_[Si_6_Cl_14_] and [*n*Bu_4_N]_2_[Si_7_Cl_16_] (Fig. 5 ▸) can be observed *via* ^29^Si NMR spectroscopy (for the NMR spectrum see Fig. S1 in the Supporting information), while [Et_4_N]_2_[Si_5_Cl_12_] is not soluble. For comparison, a ^29^Si CP/MAS NMR spectrum of single crystals of [*n*Bu_4_N]_2_[Si_5_Cl_12_] was recorded (Fig. S2 in the Supporting information).

## Refinement details   

Crystal data, data collection and structure refinement details are summarized in Table 4 ▸. H atoms were refined using a riding model, with C_meth­yl_—H = 0.98 Å or C_methyl­ene_—H = 0.99 Å and with *U*_iso_(H) = 1.5*U*_eq_(C_meth­yl_) or 1.2*U*_eq_(C).

The Cl atoms of the di­chloro­methane solvent mol­ecule in (I) have rather large displacement ellipsoids, but since no valid disorder model for splitting this mol­ecule could be found, refinement with enlarged ADPs was preferred. In (II), atoms N2 and N3 are located on centres of inversion. As a result, the ethyl­ene chains are disordered over equally occupied orientations.

## Supplementary Material

Crystal structure: contains datablock(s) I, II, global. DOI: 10.1107/S2056989017016310/hb7717sup1.cif

Structure factors: contains datablock(s) I. DOI: 10.1107/S2056989017016310/hb7717Isup2.hkl

Structure factors: contains datablock(s) II. DOI: 10.1107/S2056989017016310/hb7717IIsup3.hkl

Fig. S1. 29Si NMR spectra. DOI: 10.1107/S2056989017016310/hb7717sup4.pdf

Fig. S2. 29Si CP/MAS NMR spectrum. DOI: 10.1107/S2056989017016310/hb7717sup5.pdf

CCDC references: 1585194, 1585193

Additional supporting information:  crystallographic information; 3D view; checkCIF report

## Figures and Tables

**Table 1:** Hydrogen-bond geometry (Å, °) for (I)

*D*—H⋯*A*	*D*—H	H⋯*A*	*D*⋯*A*	*D*—H⋯*A*
C1—H1*B*⋯Cl1	0.99	2.88	3.686 (7)	139
C2—H2*A*⋯Cl31^i^	0.99	2.89	3.596 (8)	129
C5—H5*A*⋯Cl22^ii^	0.99	2.99	3.945 (7)	163
C9—H9*B*⋯Cl1	0.99	2.91	3.652 (7)	132
C1*L*—H1*L*1⋯Cl12^ii^	0.99	2.96	3.528 (13)	119

Symmetry codes: (i) 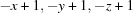; (ii) 
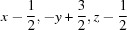
.

**Table 2:** Hydrogen-bond geometry (Å, °) for (II)

*D*—H⋯*A*	*D*—H	H⋯*A*	*D*⋯*A*	*D*—H⋯*A*
C1—H1*B*⋯Cl42	0.99	2.99	3.829 (3)	144
C2—H2*C*⋯Cl2	0.98	2.95	3.753 (3)	139
C3—H3*A*⋯Cl52^i^	0.99	2.79	3.643 (3)	144
C3—H3*B*⋯Cl2*B* ^ii^	0.99	2.98	3.804 (3)	142
C5—H5*B*⋯Cl22^iii^	0.99	2.89	3.850 (3)	165
C6—H6*B*⋯Cl21^iii^	0.98	2.86	3.630 (3)	136
C7—H7*A*⋯Cl2	0.99	2.86	3.394 (2)	115
C22—H22*C*⋯Cl51^iv^	0.98	2.89	3.847 (4)	165
C22—H22*E*⋯Cl41	0.98	2.90	3.859 (3)	165
C23—H23*B*⋯Cl1^iv^	0.99	2.98	3.465 (4)	111
C23′—H23*C*⋯Cl42	0.99	2.87	3.497 (4)	122
C24—H24*C*⋯Cl41	0.98	2.84	3.793 (3)	164
C24—H24*E*⋯Cl51^iv^	0.98	2.81	3.771 (3)	165
C24—H24*F*⋯Cl2*A* ^iv^	0.98	2.92	3.778 (3)	147
C31′—H31*C*⋯Cl31	0.99	2.95	3.434 (5)	111
C32—H32*F*⋯Cl21^v^	0.98	2.76	3.584 (4)	142
C33—H33*A*⋯Cl32^vi^	0.99	2.94	3.515 (4)	118
C33′—H33*D*⋯Cl41	0.99	2.98	3.630 (5)	124
C34—H34*A*⋯Cl1*B* ^vii^	0.98	2.93	3.556 (3)	123
C34—H34*C*⋯Cl2*B*	0.98	2.89	3.716 (4)	142
C34—H34*A*⋯Cl1*B* ^vii^	0.98	2.93	3.556 (3)	123
C1*L*—H1*L*1⋯Cl12^i^	0.99	2.90	3.421 (3)	114
C2*L*—H2*L*2⋯Cl41	0.99	2.96	3.465 (3)	113

Symmetry codes: (i) 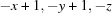; (ii) 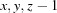; (iii) 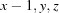; (iv) 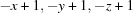; (v) 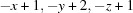; (vi) 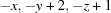; (vii) 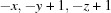.

**Table 3:** Mean values (Å) of Si—Si, Si—Cl bond lengths and Cl/N⋯*Cg* contacts in the title compounds and related structures *X* = Cl for (I) and (II) and *X* = N for ELAFON and ELAFIH. The row for Si_6_Cl_12_ contains data for dichloride dodeca­chloro­hexa­silanes (Tillmann, Lerner & Bolte, 2015 ▸).

Structure	Si—Si	Si—Cl	*X*⋯*Cg*
(I)	2.342	2.092	2.143
(II)	2.344	2.092	2.151
ELAFON	2.363	2.049	2.174
ELAFIH	2.348	2.036	2.234
Si_6_Cl_12_	2.322	2.078	1.90

**Table 4:** Experimental details

	(I)	(II)
Crystal data		
Chemical formula	2C_16_H_36_N^+^·2Cl^−^·Si_5_Cl_10_·2CH_2_Cl_2_	2C_8_H_20_N^+^·2Cl^−^·Si_5_Cl_10_·2CH_2_Cl_2_
*M*_r_	1220.61	996.20
Crystal system, space group	Monoclinic, *C*2/*c*	Triclinic, *P* 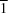
Temperature (K)	173	173
*a*, *b*, *c* (Å)	20.9091 (15), 15.7423 (7), 19.8734 (16)	10.3596 (4), 13.9612 (5), 16.0205 (6)
α, β, γ (°)	90, 112.451 (6), 90	89.959 (3), 72.484 (3), 79.534 (3)
*V* (Å^3^)	6045.7 (7)	2169.29 (15)
*Z*	4	2
Radiation type	Mo *K*α	Mo *K*α
μ (mm^−1^)	0.85	1.17
Crystal size (mm)	0.27 × 0.16 × 0.12	0.23 × 0.23 × 0.20

Data collection		
Diffractometer	Stoe IPDS II two-circle	Stoe IPDS II two-circle
Absorption correction	Multi-scan (*X-AREA*; Stoe & Cie, 2001 ▸)	Multi-scan (*X-AREA*; Stoe & Cie, 2001 ▸)
*T*_min_, *T*_max_	0.543, 1.000	0.408, 1.000
No. of measured, independent and observed [*I* > 2σ(*I*)] reflections	32650, 5699, 4428	62962, 13044, 11976
*R*_int_	0.060	0.043
(sin θ/λ)_max_ (Å^−1^)	0.612	0.715

Refinement		
*R*[*F* ^2^ > 2σ(*F* ^2^)], *wR*(*F* ^2^), *S*	0.103, 0.199, 1.15	0.048, 0.131, 1.08
No. of reflections	5699	13044
No. of parameters	258	409
H-atom treatment	H-atom parameters constrained	H-atom parameters constrained
Δρ_max_, Δρ_min_ (e Å^−3^)	1.50, −1.46	0.87, −0.84

Computer programs: *X-AREA* (Stoe & Cie, 2001 ▸), *XP* in *SHELXTL-Plus* and *SHELXS97* (Sheldrick, 2008 ▸), *SHELXL2014* (Sheldrick, 2015 ▸) and *publCIF* (Westrip, 2010 ▸).

## supplementary crystallographic information

### Bis(tetra-n-butylammonium) dichloride decachlorocyclopentasilane dichloromethane disolvate (I) Crystal data

**Table:**

2C_16_H_36_N^+^·2Cl^−^·Si_5_Cl_10_·2CH_2_Cl_2_	*F*(000) = 2544
*M**_r_* = 1220.61	*D*_x_ = 1.341 Mg m^−^^3^
Monoclinic, *C*2/*c*	Mo *K*α radiation, λ = 0.71073 Å
*a* = 20.9091 (15) Å	Cell parameters from 30920 reflections
*b* = 15.7423 (7) Å	θ = 3.3–25.8°
*c* = 19.8734 (16) Å	µ = 0.85 mm^−^^1^
β = 112.451 (6)°	*T* = 173 K
*V* = 6045.7 (7) Å^3^	Needle, colourless
*Z* = 4	0.27 × 0.16 × 0.12 mm

### Bis(tetra-n-butylammonium) dichloride decachlorocyclopentasilane dichloromethane disolvate (I) Data collection

**Table:**

Stoe IPDS II two-circle diffractometer	4428 reflections with *I* > 2σ(*I*)
Radiation source: Genix 3D IµS microfocus X-ray source	*R*_int_ = 0.060
ω scans	θ_max_ = 25.8°, θ_min_ = 3.3°
Absorption correction: multi-scan (X-AREA; Stoe & Cie, 2001)	*h* = −25→25
*T*_min_ = 0.543, *T*_max_ = 1.000	*k* = −18→19
32650 measured reflections	*l* = −24→24
5699 independent reflections	

### Bis(tetra-n-butylammonium) dichloride decachlorocyclopentasilane dichloromethane disolvate (I) Refinement

**Table:**

Refinement on *F*^2^	0 restraints
Least-squares matrix: full	Hydrogen site location: inferred from neighbouring sites
*R*[*F*^2^ > 2σ(*F*^2^)] = 0.103	H-atom parameters constrained
*wR*(*F*^2^) = 0.199	*w* = 1/[σ^2^(*F*_o_^2^) + (0.0209*P*)^2^ + 98.7944*P*] where *P* = (*F*_o_^2^ + 2*F*_c_^2^)/3
*S* = 1.15	(Δ/σ)_max_ < 0.001
5699 reflections	Δρ_max_ = 1.50 e Å^−^^3^
258 parameters	Δρ_min_ = −1.46 e Å^−^^3^

### Bis(tetra-n-butylammonium) dichloride decachlorocyclopentasilane dichloromethane disolvate (I) Special details

**Table:**

Geometry. All esds (except the esd in the dihedral angle between two l.s. planes) are estimated using the full covariance matrix. The cell esds are taken into account individually in the estimation of esds in distances, angles and torsion angles; correlations between esds in cell parameters are only used when they are defined by crystal symmetry. An approximate (isotropic) treatment of cell esds is used for estimating esds involving l.s. planes.

### Bis(tetra-n-butylammonium) dichloride decachlorocyclopentasilane dichloromethane disolvate (I) Fractional atomic coordinates and isotropic or equivalent isotropic displacement parameters (Å^2^)

**Table:**

	*x*	*y*	*z*	*U*_iso_*/*U*_eq_	
Cl1	0.44920 (9)	0.65838 (13)	0.63371 (9)	0.0392 (4)	
Si1	0.55353 (9)	0.75962 (13)	0.74394 (10)	0.0312 (4)	
Si2	0.58692 (10)	0.61853 (14)	0.73977 (10)	0.0329 (4)	
Si3	0.5000	0.53144 (19)	0.7500	0.0368 (7)	
Cl11	0.55030 (11)	0.83349 (15)	0.65484 (12)	0.0536 (5)	
Cl12	0.62942 (10)	0.82642 (15)	0.82814 (12)	0.0535 (6)	
Cl21	0.60720 (11)	0.58866 (16)	0.64782 (11)	0.0534 (6)	
Cl22	0.68493 (9)	0.59548 (15)	0.82099 (11)	0.0499 (5)	
Cl31	0.46207 (13)	0.44593 (15)	0.66352 (15)	0.0667 (7)	
N1	0.3868 (3)	0.7205 (4)	0.4082 (3)	0.0278 (12)	
C1	0.4614 (3)	0.6993 (5)	0.4564 (4)	0.0309 (15)	
H1A	0.4668	0.6368	0.4578	0.037*	
H1B	0.4699	0.7185	0.5065	0.037*	
C2	0.5172 (4)	0.7380 (5)	0.4335 (4)	0.0373 (17)	
H2A	0.5102	0.7188	0.3838	0.045*	
H2B	0.5135	0.8008	0.4329	0.045*	
C3	0.5881 (4)	0.7115 (6)	0.4859 (4)	0.047 (2)	
H3A	0.5894	0.6489	0.4913	0.056*	
H3B	0.5969	0.7367	0.5343	0.056*	
C4	0.6455 (4)	0.7395 (6)	0.4601 (6)	0.060 (3)	
H4A	0.6905	0.7210	0.4956	0.090*	
H4B	0.6375	0.7137	0.4127	0.090*	
H4C	0.6450	0.8015	0.4557	0.090*	
C5	0.3758 (4)	0.8156 (5)	0.3999 (4)	0.0351 (16)	
H5A	0.3258	0.8265	0.3728	0.042*	
H5B	0.4007	0.8374	0.3700	0.042*	
C6	0.3994 (4)	0.8659 (5)	0.4706 (4)	0.0440 (19)	
H6A	0.3744	0.8457	0.5010	0.053*	
H6B	0.4495	0.8568	0.4981	0.053*	
C7	0.3852 (4)	0.9597 (5)	0.4545 (5)	0.055 (2)	
H7A	0.3348	0.9685	0.4288	0.066*	
H7B	0.4082	0.9787	0.4219	0.066*	
C8	0.4110 (5)	1.0135 (6)	0.5234 (6)	0.078 (3)	
H8A	0.4008	1.0734	0.5104	0.116*	
H8B	0.3877	0.9957	0.5554	0.116*	
H8C	0.4611	1.0059	0.5485	0.116*	
C9	0.3385 (3)	0.6853 (5)	0.4418 (4)	0.0303 (15)	
H9A	0.2910	0.7045	0.4120	0.036*	
H9B	0.3513	0.7107	0.4908	0.036*	
C10	0.3372 (4)	0.5894 (5)	0.4494 (4)	0.0396 (17)	
H10A	0.3273	0.5623	0.4015	0.047*	
H10B	0.3830	0.5693	0.4835	0.047*	
C11	0.2824 (4)	0.5642 (5)	0.4778 (4)	0.044 (2)	
H11A	0.2364	0.5815	0.4420	0.053*	
H11B	0.2906	0.5951	0.5237	0.053*	
C12	0.2818 (4)	0.4698 (6)	0.4915 (5)	0.059 (2)	
H12A	0.2455	0.4567	0.5097	0.088*	
H12B	0.2727	0.4389	0.4459	0.088*	
H12C	0.3268	0.4525	0.5276	0.088*	
C13	0.3714 (4)	0.6817 (5)	0.3333 (3)	0.0348 (16)	
H13A	0.4026	0.7085	0.3125	0.042*	
H13B	0.3834	0.6206	0.3400	0.042*	
C14	0.2978 (4)	0.6896 (6)	0.2779 (4)	0.0412 (18)	
H14A	0.2839	0.7501	0.2711	0.049*	
H14B	0.2657	0.6589	0.2953	0.049*	
C15	0.2940 (4)	0.6515 (7)	0.2051 (4)	0.057 (2)	
H15A	0.3294	0.6789	0.1907	0.068*	
H15B	0.3049	0.5901	0.2118	0.068*	
C16	0.2249 (5)	0.6627 (9)	0.1459 (5)	0.083 (4)	
H16A	0.2251	0.6375	0.1009	0.124*	
H16B	0.1898	0.6346	0.1594	0.124*	
H16C	0.2143	0.7234	0.1383	0.124*	
C1L	0.1235 (8)	0.4569 (8)	0.2807 (9)	0.110 (5)	
H1L1	0.1317	0.4857	0.3275	0.132*	
H1L2	0.0904	0.4924	0.2416	0.132*	
Cl1L	0.19998 (18)	0.4539 (3)	0.2681 (2)	0.1200 (15)	
Cl2L	0.0881 (5)	0.3685 (5)	0.2810 (5)	0.294 (6)	

### Bis(tetra-n-butylammonium) dichloride decachlorocyclopentasilane dichloromethane disolvate (I) Atomic displacement parameters (Å^2^)

**Table:**

	*U*^11^	*U*^22^	*U*^33^	*U*^12^	*U*^13^	*U*^23^
Cl1	0.0321 (9)	0.0584 (12)	0.0230 (8)	−0.0037 (9)	0.0059 (7)	−0.0024 (8)
Si1	0.0233 (9)	0.0364 (11)	0.0327 (10)	−0.0031 (8)	0.0092 (8)	−0.0029 (8)
Si2	0.0281 (9)	0.0417 (12)	0.0302 (10)	0.0050 (9)	0.0124 (8)	−0.0018 (9)
Si3	0.0373 (15)	0.0330 (16)	0.0421 (16)	0.000	0.0174 (13)	0.000
Cl11	0.0498 (11)	0.0575 (14)	0.0576 (13)	−0.0087 (10)	0.0251 (10)	0.0109 (11)
Cl12	0.0345 (10)	0.0572 (14)	0.0600 (13)	−0.0137 (9)	0.0082 (9)	−0.0193 (11)
Cl21	0.0526 (12)	0.0739 (15)	0.0441 (11)	0.0101 (11)	0.0302 (10)	−0.0056 (10)
Cl22	0.0276 (9)	0.0708 (15)	0.0470 (11)	0.0139 (9)	0.0095 (8)	0.0056 (10)
Cl31	0.0663 (15)	0.0501 (13)	0.0859 (18)	−0.0109 (12)	0.0315 (13)	−0.0313 (13)
N1	0.027 (3)	0.032 (3)	0.028 (3)	0.003 (2)	0.014 (2)	0.002 (2)
C1	0.025 (3)	0.036 (4)	0.030 (4)	0.002 (3)	0.009 (3)	0.000 (3)
C2	0.044 (4)	0.035 (4)	0.039 (4)	−0.006 (3)	0.023 (3)	−0.005 (3)
C3	0.030 (4)	0.065 (6)	0.045 (4)	−0.006 (4)	0.014 (3)	−0.011 (4)
C4	0.045 (5)	0.054 (6)	0.096 (7)	−0.011 (4)	0.043 (5)	−0.018 (5)
C5	0.032 (4)	0.035 (4)	0.040 (4)	0.009 (3)	0.015 (3)	0.003 (3)
C6	0.052 (5)	0.039 (4)	0.046 (4)	0.001 (4)	0.024 (4)	−0.007 (4)
C7	0.042 (5)	0.038 (5)	0.082 (7)	0.005 (4)	0.020 (4)	−0.012 (4)
C8	0.063 (6)	0.052 (6)	0.116 (9)	−0.004 (5)	0.033 (6)	−0.034 (6)
C9	0.024 (3)	0.042 (4)	0.025 (3)	0.000 (3)	0.010 (3)	0.003 (3)
C10	0.039 (4)	0.043 (4)	0.041 (4)	−0.002 (4)	0.019 (3)	0.003 (4)
C11	0.028 (4)	0.057 (5)	0.049 (5)	0.001 (4)	0.016 (3)	0.016 (4)
C12	0.043 (5)	0.059 (6)	0.079 (7)	−0.005 (4)	0.027 (5)	0.019 (5)
C13	0.040 (4)	0.043 (4)	0.025 (3)	0.004 (3)	0.017 (3)	−0.002 (3)
C14	0.036 (4)	0.055 (5)	0.030 (4)	−0.003 (4)	0.010 (3)	−0.005 (3)
C15	0.044 (5)	0.082 (7)	0.045 (5)	0.005 (5)	0.018 (4)	−0.005 (5)
C16	0.056 (6)	0.150 (12)	0.035 (5)	0.004 (7)	0.009 (4)	−0.019 (6)
C1L	0.143 (13)	0.077 (9)	0.149 (13)	0.017 (9)	0.100 (11)	−0.003 (9)
Cl1L	0.087 (2)	0.168 (4)	0.114 (3)	−0.022 (2)	0.048 (2)	−0.065 (3)
Cl2L	0.471 (12)	0.237 (7)	0.350 (10)	−0.225 (8)	0.355 (10)	−0.183 (7)

### Bis(tetra-n-butylammonium) dichloride decachlorocyclopentasilane dichloromethane disolvate (I) Geometric parameters (Å, º)

**Table:**

Si1—Cl12	2.097 (3)	C7—H7A	0.9900
Si1—Cl11	2.098 (3)	C7—H7B	0.9900
Si1—Si2	2.339 (3)	C8—H8A	0.9800
Si1—Si1^i^	2.341 (4)	C8—H8B	0.9800
Si2—Cl21	2.081 (3)	C8—H8C	0.9800
Si2—Cl22	2.100 (3)	C9—C10	1.519 (10)
Si2—Si3	2.347 (3)	C9—H9A	0.9900
Si3—Cl31	2.086 (3)	C9—H9B	0.9900
Si3—Cl31^i^	2.086 (3)	C10—C11	1.509 (9)
Si3—Si2^i^	2.347 (3)	C10—H10A	0.9900
N1—C9	1.513 (8)	C10—H10B	0.9900
N1—C5	1.515 (9)	C11—C12	1.513 (12)
N1—C1	1.523 (8)	C11—H11A	0.9900
N1—C13	1.525 (8)	C11—H11B	0.9900
C1—C2	1.531 (9)	C12—H12A	0.9800
C1—H1A	0.9900	C12—H12B	0.9800
C1—H1B	0.9900	C12—H12C	0.9800
C2—C3	1.510 (10)	C13—C14	1.518 (10)
C2—H2A	0.9900	C13—H13A	0.9900
C2—H2B	0.9900	C13—H13B	0.9900
C3—C4	1.538 (10)	C14—C15	1.539 (10)
C3—H3A	0.9900	C14—H14A	0.9900
C3—H3B	0.9900	C14—H14B	0.9900
C4—H4A	0.9800	C15—C16	1.485 (12)
C4—H4B	0.9800	C15—H15A	0.9900
C4—H4C	0.9800	C15—H15B	0.9900
C5—C6	1.521 (10)	C16—H16A	0.9800
C5—H5A	0.9900	C16—H16B	0.9800
C5—H5B	0.9900	C16—H16C	0.9800
C6—C7	1.516 (11)	C1L—Cl2L	1.578 (14)
C6—H6A	0.9900	C1L—Cl1L	1.711 (13)
C6—H6B	0.9900	C1L—H1L1	0.9900
C7—C8	1.522 (13)	C1L—H1L2	0.9900
			
Cl12—Si1—Cl11	99.57 (12)	C6—C7—H7B	109.2
Cl12—Si1—Si2	111.06 (11)	C8—C7—H7B	109.2
Cl11—Si1—Si2	114.22 (11)	H7A—C7—H7B	107.9
Cl12—Si1—Si1^i^	112.32 (12)	C7—C8—H8A	109.5
Cl11—Si1—Si1^i^	111.28 (12)	C7—C8—H8B	109.5
Si2—Si1—Si1^i^	108.28 (6)	H8A—C8—H8B	109.5
Cl21—Si2—Cl22	99.76 (11)	C7—C8—H8C	109.5
Cl21—Si2—Si1	114.16 (12)	H8A—C8—H8C	109.5
Cl22—Si2—Si1	110.71 (12)	H8B—C8—H8C	109.5
Cl21—Si2—Si3	111.61 (12)	N1—C9—C10	116.6 (6)
Cl22—Si2—Si3	113.15 (11)	N1—C9—H9A	108.1
Si1—Si2—Si3	107.47 (10)	C10—C9—H9A	108.1
Cl31—Si3—Cl31^i^	99.6 (2)	N1—C9—H9B	108.1
Cl31—Si3—Si2	111.65 (9)	C10—C9—H9B	108.1
Cl31^i^—Si3—Si2	112.64 (9)	H9A—C9—H9B	107.3
Cl31—Si3—Si2^i^	112.64 (9)	C11—C10—C9	110.0 (6)
Cl31^i^—Si3—Si2^i^	111.65 (9)	C11—C10—H10A	109.7
Si2—Si3—Si2^i^	108.50 (16)	C9—C10—H10A	109.7
C9—N1—C5	108.1 (5)	C11—C10—H10B	109.7
C9—N1—C1	109.8 (5)	C9—C10—H10B	109.7
C5—N1—C1	111.2 (5)	H10A—C10—H10B	108.2
C9—N1—C13	110.9 (5)	C10—C11—C12	112.6 (7)
C5—N1—C13	108.8 (5)	C10—C11—H11A	109.1
C1—N1—C13	108.0 (5)	C12—C11—H11A	109.1
N1—C1—C2	116.1 (6)	C10—C11—H11B	109.1
N1—C1—H1A	108.3	C12—C11—H11B	109.1
C2—C1—H1A	108.3	H11A—C11—H11B	107.8
N1—C1—H1B	108.3	C11—C12—H12A	109.5
C2—C1—H1B	108.3	C11—C12—H12B	109.5
H1A—C1—H1B	107.4	H12A—C12—H12B	109.5
C3—C2—C1	110.1 (6)	C11—C12—H12C	109.5
C3—C2—H2A	109.6	H12A—C12—H12C	109.5
C1—C2—H2A	109.6	H12B—C12—H12C	109.5
C3—C2—H2B	109.6	C14—C13—N1	116.9 (6)
C1—C2—H2B	109.6	C14—C13—H13A	108.1
H2A—C2—H2B	108.1	N1—C13—H13A	108.1
C2—C3—C4	112.1 (7)	C14—C13—H13B	108.1
C2—C3—H3A	109.2	N1—C13—H13B	108.1
C4—C3—H3A	109.2	H13A—C13—H13B	107.3
C2—C3—H3B	109.2	C13—C14—C15	108.9 (6)
C4—C3—H3B	109.2	C13—C14—H14A	109.9
H3A—C3—H3B	107.9	C15—C14—H14A	109.9
C3—C4—H4A	109.5	C13—C14—H14B	109.9
C3—C4—H4B	109.5	C15—C14—H14B	109.9
H4A—C4—H4B	109.5	H14A—C14—H14B	108.3
C3—C4—H4C	109.5	C16—C15—C14	112.5 (7)
H4A—C4—H4C	109.5	C16—C15—H15A	109.1
H4B—C4—H4C	109.5	C14—C15—H15A	109.1
N1—C5—C6	115.6 (6)	C16—C15—H15B	109.1
N1—C5—H5A	108.4	C14—C15—H15B	109.1
C6—C5—H5A	108.4	H15A—C15—H15B	107.8
N1—C5—H5B	108.4	C15—C16—H16A	109.5
C6—C5—H5B	108.4	C15—C16—H16B	109.5
H5A—C5—H5B	107.4	H16A—C16—H16B	109.5
C7—C6—C5	110.1 (7)	C15—C16—H16C	109.5
C7—C6—H6A	109.6	H16A—C16—H16C	109.5
C5—C6—H6A	109.6	H16B—C16—H16C	109.5
C7—C6—H6B	109.6	Cl2L—C1L—Cl1L	116.2 (8)
C5—C6—H6B	109.6	Cl2L—C1L—H1L1	108.2
H6A—C6—H6B	108.2	Cl1L—C1L—H1L1	108.2
C6—C7—C8	112.3 (8)	Cl2L—C1L—H1L2	108.2
C6—C7—H7A	109.2	Cl1L—C1L—H1L2	108.2
C8—C7—H7A	109.2	H1L1—C1L—H1L2	107.4
			
C9—N1—C1—C2	−174.3 (6)	C5—N1—C9—C10	175.4 (6)
C5—N1—C1—C2	−54.8 (7)	C1—N1—C9—C10	−63.2 (7)
C13—N1—C1—C2	64.6 (7)	C13—N1—C9—C10	56.1 (8)
N1—C1—C2—C3	−179.8 (6)	N1—C9—C10—C11	−175.2 (6)
C1—C2—C3—C4	172.9 (7)	C9—C10—C11—C12	−176.1 (7)
C9—N1—C5—C6	68.4 (7)	C9—N1—C13—C14	55.2 (8)
C1—N1—C5—C6	−52.2 (7)	C5—N1—C13—C14	−63.6 (8)
C13—N1—C5—C6	−171.0 (6)	C1—N1—C13—C14	175.6 (6)
N1—C5—C6—C7	−179.9 (6)	N1—C13—C14—C15	176.8 (7)
C5—C6—C7—C8	−177.3 (7)	C13—C14—C15—C16	−175.4 (9)

Symmetry code: (i) −*x*+1, *y*, −*z*+3/2.

### Bis(tetra-n-butylammonium) dichloride decachlorocyclopentasilane dichloromethane disolvate (I) Hydrogen-bond geometry (Å, º)

**Table:**

*D*—H···*A*	*D*—H	H···*A*	*D*···*A*	*D*—H···*A*
C1—H1*B*···Cl1	0.99	2.88	3.686 (7)	139
C2—H2*A*···Cl31^ii^	0.99	2.89	3.596 (8)	129
C5—H5*A*···Cl22^iii^	0.99	2.99	3.945 (7)	163
C9—H9*B*···Cl1	0.99	2.91	3.652 (7)	132
C1*L*—H1*L*1···Cl12^iii^	0.99	2.96	3.528 (13)	119

Symmetry codes: (ii) −*x*+1, −*y*+1, −*z*+1; (iii) *x*−1/2, −*y*+3/2, *z*−1/2.

### Bis(tetraethylammonium) dichloride decachlorocyclopentasilane dichloromethane disolvate (II) Crystal data

**Table:**

2C_8_H_20_N^+^·2Cl^−^·Si_5_Cl_10_·2CH_2_Cl_2_	*Z* = 2
*M**_r_* = 996.20	*F*(000) = 1016
Triclinic, *P*1	*D*_x_ = 1.525 Mg m^−^^3^
*a* = 10.3596 (4) Å	Mo *K*α radiation, λ = 0.71073 Å
*b* = 13.9612 (5) Å	Cell parameters from 153722 reflections
*c* = 16.0205 (6) Å	θ = 3.3–30.8°
α = 89.959 (3)°	µ = 1.17 mm^−^^1^
β = 72.484 (3)°	*T* = 173 K
γ = 79.534 (3)°	Block, colourless
*V* = 2169.29 (15) Å^3^	0.23 × 0.23 × 0.20 mm

### Bis(tetraethylammonium) dichloride decachlorocyclopentasilane dichloromethane disolvate (II) Data collection

**Table:**

Stoe IPDS II two-circle diffractometer	11976 reflections with *I* > 2σ(*I*)
ω scans	*R*_int_ = 0.043
Absorption correction: multi-scan (X-AREA; Stoe & Cie, 2001)	θ_max_ = 30.5°, θ_min_ = 3.4°
*T*_min_ = 0.408, *T*_max_ = 1.000	*h* = −14→14
62962 measured reflections	*k* = −19→19
13044 independent reflections	*l* = −22→22

### Bis(tetraethylammonium) dichloride decachlorocyclopentasilane dichloromethane disolvate (II) Refinement

**Table:**

Refinement on *F*^2^	0 restraints
Least-squares matrix: full	Hydrogen site location: inferred from neighbouring sites
*R*[*F*^2^ > 2σ(*F*^2^)] = 0.048	H-atom parameters constrained
*wR*(*F*^2^) = 0.131	*w* = 1/[σ^2^(*F*_o_^2^) + (0.0683*P*)^2^ + 1.6519*P*] where *P* = (*F*_o_^2^ + 2*F*_c_^2^)/3
*S* = 1.08	(Δ/σ)_max_ = 0.001
13044 reflections	Δρ_max_ = 0.87 e Å^−^^3^
409 parameters	Δρ_min_ = −0.84 e Å^−^^3^

### Bis(tetraethylammonium) dichloride decachlorocyclopentasilane dichloromethane disolvate (II) Special details

**Table:**

Geometry. All esds (except the esd in the dihedral angle between two l.s. planes) are estimated using the full covariance matrix. The cell esds are taken into account individually in the estimation of esds in distances, angles and torsion angles; correlations between esds in cell parameters are only used when they are defined by crystal symmetry. An approximate (isotropic) treatment of cell esds is used for estimating esds involving l.s. planes.

### Bis(tetraethylammonium) dichloride decachlorocyclopentasilane dichloromethane disolvate (II) Fractional atomic coordinates and isotropic or equivalent isotropic displacement parameters (Å^2^)

**Table:**

	*x*	*y*	*z*	*U*_iso_*/*U*_eq_	Occ. (<1)
Cl1	0.68362 (5)	0.71247 (4)	0.37887 (3)	0.03357 (10)	
Cl2	0.55394 (6)	0.71721 (4)	0.14756 (3)	0.03425 (11)	
Si1	0.79603 (6)	0.63165 (4)	0.19539 (4)	0.03316 (12)	
Si2	0.74595 (6)	0.80223 (4)	0.20699 (4)	0.02939 (11)	
Si3	0.51729 (5)	0.85241 (4)	0.29623 (3)	0.02570 (10)	
Si4	0.42527 (5)	0.71172 (4)	0.33588 (3)	0.02431 (10)	
Si5	0.59853 (6)	0.57465 (4)	0.27696 (4)	0.02724 (11)	
Cl11	0.97197 (6)	0.57714 (6)	0.23162 (6)	0.0628 (2)	
Cl12	0.86579 (9)	0.57414 (5)	0.06642 (4)	0.0632 (2)	
Cl21	0.88627 (7)	0.86092 (6)	0.25209 (5)	0.05179 (16)	
Cl22	0.78501 (6)	0.86177 (4)	0.08273 (4)	0.04303 (13)	
Cl31	0.49581 (7)	0.94164 (4)	0.40573 (4)	0.04399 (13)	
Cl32	0.39997 (6)	0.94686 (4)	0.23306 (4)	0.04020 (12)	
Cl41	0.33929 (5)	0.71094 (4)	0.47227 (3)	0.03436 (10)	
Cl42	0.24659 (5)	0.71209 (4)	0.29964 (4)	0.03703 (11)	
Cl51	0.63586 (6)	0.47736 (4)	0.36927 (4)	0.04057 (12)	
Cl52	0.53223 (8)	0.48498 (4)	0.20041 (4)	0.04831 (15)	
N1	0.27041 (18)	0.79721 (13)	−0.00541 (11)	0.0299 (3)	
C1	0.2204 (4)	0.7299 (2)	0.06657 (18)	0.0551 (7)	
H1A	0.1238	0.7262	0.0717	0.066*	
H1B	0.2206	0.7590	0.1228	0.066*	
C2	0.3050 (4)	0.62647 (19)	0.0530 (2)	0.0551 (7)	
H2A	0.2656	0.5882	0.1024	0.083*	
H2B	0.3034	0.5959	−0.0016	0.083*	
H2C	0.4004	0.6288	0.0497	0.083*	
C3	0.2867 (3)	0.7533 (2)	−0.09498 (16)	0.0438 (5)	
H3A	0.3641	0.6966	−0.1089	0.053*	
H3B	0.3126	0.8021	−0.1387	0.053*	
C4	0.1610 (4)	0.7201 (3)	−0.1052 (3)	0.0648 (9)	
H4A	0.1816	0.6928	−0.1651	0.097*	
H4B	0.1357	0.6701	−0.0636	0.097*	
H4C	0.0842	0.7759	−0.0934	0.097*	
C5	0.1640 (3)	0.89268 (19)	0.0117 (2)	0.0470 (6)	
H5A	0.1863	0.9314	−0.0407	0.056*	
H5B	0.0721	0.8767	0.0192	0.056*	
C6	0.1555 (3)	0.9554 (2)	0.0910 (2)	0.0545 (7)	
H6A	0.0852	1.0145	0.0967	0.082*	
H6B	0.1306	0.9187	0.1438	0.082*	
H6C	0.2451	0.9736	0.0837	0.082*	
C7	0.4085 (3)	0.81784 (18)	−0.00349 (18)	0.0412 (5)	
H7A	0.3980	0.8424	0.0566	0.049*	
H7B	0.4756	0.7554	−0.0155	0.049*	
C8	0.4683 (3)	0.8892 (2)	−0.0662 (2)	0.0517 (6)	
H8A	0.5568	0.8970	−0.0594	0.078*	
H8B	0.4826	0.8651	−0.1264	0.078*	
H8C	0.4048	0.9523	−0.0540	0.078*	
N2	0.0000	0.5000	0.5000	0.0260 (4)	
C21	−0.0005 (4)	0.6091 (3)	0.5117 (3)	0.0331 (8)	0.5
H21A	−0.0855	0.6483	0.5040	0.040*	0.5
H21B	0.0800	0.6270	0.4675	0.040*	0.5
C21'	0.0128 (5)	0.5139 (3)	0.5909 (3)	0.0350 (8)	0.5
H21C	0.1013	0.4755	0.5941	0.042*	0.5
H21D	−0.0633	0.4912	0.6352	0.042*	0.5
C22	0.0063 (3)	0.6300 (3)	0.6105 (2)	0.0638 (9)	
H22A	0.0061	0.6994	0.6197	0.096*	0.5
H22B	−0.0740	0.6122	0.6536	0.096*	0.5
H22C	0.0908	0.5910	0.6173	0.096*	0.5
H22D	0.0144	0.6403	0.6690	0.096*	0.5
H22E	0.0823	0.6518	0.5665	0.096*	0.5
H22F	−0.0818	0.6675	0.6075	0.096*	0.5
C23	0.1319 (4)	0.4394 (3)	0.5085 (3)	0.0294 (7)	0.5
H23A	0.1285	0.3693	0.5040	0.035*	0.5
H23B	0.1411	0.4544	0.5666	0.035*	0.5
C23'	0.1182 (4)	0.5289 (3)	0.4300 (3)	0.0321 (7)	0.5
H23C	0.1196	0.5986	0.4398	0.039*	0.5
H23D	0.1063	0.5197	0.3717	0.039*	0.5
C24	0.2624 (2)	0.4624 (2)	0.4327 (2)	0.0533 (7)	
H24A	0.3471	0.4228	0.4389	0.080*	0.5
H24B	0.2538	0.4467	0.3753	0.080*	0.5
H24C	0.2662	0.5317	0.4377	0.080*	0.5
H24D	0.3391	0.4813	0.3871	0.080*	0.5
H24E	0.2743	0.4723	0.4903	0.080*	0.5
H24F	0.2610	0.3935	0.4224	0.080*	0.5
N3	0.0000	1.0000	0.5000	0.0324 (5)	
C31	−0.0124 (7)	1.0998 (4)	0.5376 (4)	0.0522 (13)	0.5
H31A	−0.0599	1.1032	0.6015	0.063*	0.5
H31B	−0.0684	1.1477	0.5106	0.063*	0.5
C31'	0.1452 (5)	1.0189 (5)	0.4881 (4)	0.0509 (13)	0.5
H31C	0.2003	1.0076	0.4255	0.061*	0.5
H31D	0.1910	0.9729	0.5221	0.061*	0.5
C32	0.1392 (6)	1.1271 (4)	0.5199 (3)	0.0956 (17)	
H32A	0.1294	1.1930	0.5450	0.143*	0.5
H32B	0.1941	1.0803	0.5474	0.143*	0.5
H32C	0.1855	1.1246	0.4566	0.143*	0.5
H32D	0.2330	1.1379	0.5118	0.143*	0.5
H32E	0.0951	1.1727	0.4856	0.143*	0.5
H32F	0.0857	1.1380	0.5821	0.143*	0.5
C33	−0.0905 (5)	1.0176 (4)	0.5927 (3)	0.0412 (10)	0.5
H33A	−0.1850	1.0092	0.5967	0.049*	0.5
H33B	−0.0952	1.0851	0.6140	0.049*	0.5
C33'	0.0691 (5)	0.9243 (4)	0.5529 (3)	0.0459 (11)	0.5
H33C	0.1629	0.9350	0.5479	0.055*	0.5
H33D	0.0765	0.8572	0.5296	0.055*	0.5
C34	−0.0250 (4)	0.9384 (3)	0.6531 (2)	0.0724 (11)	
H34A	−0.0833	0.9490	0.7144	0.109*	0.5
H34B	−0.0212	0.8719	0.6318	0.109*	0.5
H34C	0.0683	0.9475	0.6490	0.109*	0.5
H34D	0.0162	0.8916	0.6879	0.109*	0.5
H34E	−0.0314	1.0050	0.6756	0.109*	0.5
H34F	−0.1175	0.9274	0.6574	0.109*	0.5
C1L	0.1880 (4)	0.3162 (2)	0.1144 (2)	0.0565 (7)	
H1L1	0.2330	0.2960	0.0516	0.068*	
H1L2	0.0997	0.3611	0.1200	0.068*	
Cl1A	0.29537 (8)	0.37721 (5)	0.15334 (6)	0.05663 (17)	
Cl1B	0.15522 (9)	0.21224 (6)	0.17452 (6)	0.05883 (18)	
C2L	0.4865 (4)	0.7900 (3)	0.6208 (2)	0.0642 (8)	
H2L1	0.5201	0.8228	0.5657	0.077*	
H2L2	0.4831	0.7222	0.6050	0.077*	
Cl2A	0.60192 (10)	0.78831 (6)	0.68194 (6)	0.0634 (2)	
Cl2B	0.32076 (10)	0.85079 (10)	0.67916 (6)	0.0828 (3)	

### Bis(tetraethylammonium) dichloride decachlorocyclopentasilane dichloromethane disolvate (II) Atomic displacement parameters (Å^2^)

**Table:**

	*U*^11^	*U*^22^	*U*^33^	*U*^12^	*U*^13^	*U*^23^
Cl1	0.0361 (2)	0.0429 (3)	0.0274 (2)	−0.01369 (19)	−0.01464 (17)	0.00581 (17)
Cl2	0.0424 (3)	0.0357 (2)	0.0271 (2)	−0.00201 (19)	−0.01742 (18)	−0.00062 (16)
Si1	0.0284 (2)	0.0311 (3)	0.0307 (3)	0.0042 (2)	−0.0012 (2)	0.0051 (2)
Si2	0.0258 (2)	0.0307 (3)	0.0279 (2)	−0.00452 (19)	−0.00314 (19)	0.00405 (19)
Si3	0.0266 (2)	0.0229 (2)	0.0245 (2)	−0.00328 (17)	−0.00405 (18)	−0.00117 (17)
Si4	0.0239 (2)	0.0234 (2)	0.0246 (2)	−0.00303 (17)	−0.00662 (17)	−0.00054 (16)
Si5	0.0287 (2)	0.0228 (2)	0.0295 (2)	−0.00127 (18)	−0.00988 (19)	−0.00119 (18)
Cl11	0.0288 (3)	0.0663 (4)	0.0855 (5)	0.0061 (3)	−0.0147 (3)	0.0272 (4)
Cl12	0.0766 (5)	0.0475 (3)	0.0344 (3)	0.0162 (3)	0.0127 (3)	−0.0050 (2)
Cl21	0.0423 (3)	0.0669 (4)	0.0530 (3)	−0.0301 (3)	−0.0132 (3)	0.0083 (3)
Cl22	0.0427 (3)	0.0432 (3)	0.0342 (2)	−0.0043 (2)	−0.0008 (2)	0.0144 (2)
Cl31	0.0582 (3)	0.0344 (2)	0.0360 (3)	−0.0095 (2)	−0.0090 (2)	−0.0121 (2)
Cl32	0.0410 (3)	0.0302 (2)	0.0445 (3)	0.00368 (19)	−0.0119 (2)	0.00648 (19)
Cl41	0.0336 (2)	0.0416 (3)	0.0253 (2)	−0.01096 (19)	−0.00306 (17)	0.00253 (17)
Cl42	0.0285 (2)	0.0429 (3)	0.0433 (3)	−0.00619 (18)	−0.01663 (19)	0.0021 (2)
Cl51	0.0404 (3)	0.0343 (2)	0.0516 (3)	−0.0089 (2)	−0.0198 (2)	0.0174 (2)
Cl52	0.0629 (4)	0.0337 (3)	0.0529 (3)	−0.0081 (2)	−0.0251 (3)	−0.0133 (2)
N1	0.0310 (8)	0.0304 (8)	0.0275 (7)	−0.0052 (6)	−0.0080 (6)	0.0030 (6)
C1	0.0746 (19)	0.0423 (13)	0.0395 (12)	−0.0160 (13)	−0.0016 (12)	0.0076 (10)
C2	0.085 (2)	0.0343 (12)	0.0522 (15)	−0.0151 (13)	−0.0287 (15)	0.0117 (10)
C3	0.0530 (14)	0.0456 (12)	0.0365 (11)	−0.0105 (10)	−0.0187 (10)	−0.0010 (9)
C4	0.069 (2)	0.0598 (18)	0.087 (2)	−0.0171 (15)	−0.0524 (19)	−0.0019 (16)
C5	0.0357 (11)	0.0378 (12)	0.0634 (16)	−0.0041 (9)	−0.0110 (11)	0.0002 (11)
C6	0.0512 (15)	0.0407 (13)	0.0554 (15)	−0.0079 (11)	0.0070 (12)	−0.0096 (11)
C7	0.0378 (11)	0.0392 (11)	0.0498 (13)	−0.0028 (9)	−0.0208 (10)	0.0006 (9)
C8	0.0378 (12)	0.0480 (14)	0.0643 (17)	−0.0150 (10)	−0.0042 (11)	−0.0017 (12)
N2	0.0230 (9)	0.0267 (10)	0.0268 (10)	−0.0036 (8)	−0.0063 (8)	0.0051 (8)
C21	0.0329 (18)	0.0228 (16)	0.040 (2)	−0.0042 (14)	−0.0058 (16)	0.0055 (14)
C21'	0.0330 (19)	0.043 (2)	0.0293 (17)	−0.0049 (16)	−0.0108 (15)	0.0050 (15)
C22	0.0471 (15)	0.073 (2)	0.0663 (19)	−0.0141 (14)	−0.0078 (13)	−0.0342 (16)
C23	0.0252 (16)	0.0297 (17)	0.0327 (17)	−0.0030 (13)	−0.0095 (14)	0.0056 (14)
C23'	0.0275 (17)	0.0336 (18)	0.0322 (18)	−0.0082 (14)	−0.0031 (14)	0.0067 (14)
C24	0.0240 (9)	0.0589 (16)	0.0671 (17)	−0.0074 (10)	0.0005 (10)	−0.0163 (13)
N3	0.0243 (10)	0.0345 (12)	0.0329 (11)	0.0000 (9)	−0.0037 (9)	−0.0037 (9)
C31	0.072 (4)	0.037 (2)	0.040 (2)	−0.006 (2)	−0.009 (2)	−0.0055 (19)
C31'	0.030 (2)	0.081 (4)	0.043 (3)	−0.015 (2)	−0.0107 (18)	0.013 (2)
C32	0.146 (4)	0.103 (3)	0.080 (3)	−0.084 (3)	−0.061 (3)	0.023 (2)
C33	0.034 (2)	0.049 (2)	0.0292 (18)	0.0039 (18)	0.0004 (16)	−0.0031 (17)
C33'	0.033 (2)	0.055 (3)	0.045 (2)	0.0018 (19)	−0.0118 (19)	0.009 (2)
C34	0.083 (2)	0.110 (3)	0.0381 (14)	−0.048 (2)	−0.0207 (15)	0.0216 (16)
C1L	0.0620 (17)	0.0570 (16)	0.0647 (18)	−0.0176 (14)	−0.0369 (15)	0.0150 (13)
Cl1A	0.0500 (3)	0.0452 (3)	0.0797 (5)	−0.0104 (3)	−0.0265 (3)	0.0018 (3)
Cl1B	0.0613 (4)	0.0527 (4)	0.0685 (5)	−0.0195 (3)	−0.0237 (4)	0.0092 (3)
C2L	0.0639 (19)	0.078 (2)	0.0503 (16)	0.0043 (16)	−0.0266 (14)	−0.0118 (15)
Cl2A	0.0746 (5)	0.0622 (4)	0.0588 (4)	0.0015 (4)	−0.0367 (4)	−0.0093 (3)
Cl2B	0.0612 (5)	0.1170 (9)	0.0576 (5)	0.0084 (5)	−0.0143 (4)	0.0110 (5)

### Bis(tetraethylammonium) dichloride decachlorocyclopentasilane dichloromethane disolvate (II) Geometric parameters (Å, º)

**Table:**

Si1—Cl12	2.0805 (9)	C22—H22B	0.9800
Si1—Cl11	2.0906 (9)	C22—H22C	0.9800
Si1—Si2	2.3386 (8)	C22—H22D	0.9800
Si1—Si5	2.3469 (8)	C22—H22E	0.9800
Si2—Cl21	2.0910 (8)	C22—H22F	0.9800
Si2—Cl22	2.1102 (8)	C23—C24	1.604 (5)
Si2—Si3	2.3465 (7)	C23—H23A	0.9900
Si3—Cl31	2.0849 (7)	C23—H23B	0.9900
Si3—Cl32	2.0967 (8)	C23'—C24	1.622 (5)
Si3—Si4	2.3419 (7)	C23'—H23C	0.9900
Si4—Cl41	2.0977 (7)	C23'—H23D	0.9900
Si4—Cl42	2.0993 (7)	C24—H24A	0.9800
Si4—Si5	2.3473 (7)	C24—H24B	0.9800
Si5—Cl51	2.0814 (7)	C24—H24C	0.9800
Si5—Cl52	2.0890 (8)	C24—H24D	0.9800
N1—C3	1.510 (3)	C24—H24E	0.9800
N1—C1	1.516 (3)	C24—H24F	0.9800
N1—C7	1.519 (3)	N3—C31^ii^	1.487 (5)
N1—C5	1.533 (3)	N3—C31	1.487 (5)
C1—C2	1.526 (4)	N3—C33^ii^	1.491 (4)
C1—H1A	0.9900	N3—C33	1.491 (4)
C1—H1B	0.9900	N3—C31'^ii^	1.530 (5)
C2—H2A	0.9800	N3—C31'	1.530 (5)
C2—H2B	0.9800	N3—C33'^ii^	1.561 (5)
C2—H2C	0.9800	N3—C33'	1.561 (5)
C3—C4	1.511 (4)	C31—C32	1.625 (8)
C3—H3A	0.9900	C31—H31A	0.9900
C3—H3B	0.9900	C31—H31B	0.9900
C4—H4A	0.9800	C31'—C32	1.579 (8)
C4—H4B	0.9800	C31'—H31C	0.9900
C4—H4C	0.9800	C31'—H31D	0.9900
C5—C6	1.514 (4)	C32—H32A	0.9800
C5—H5A	0.9900	C32—H32B	0.9800
C5—H5B	0.9900	C32—H32C	0.9800
C6—H6A	0.9800	C32—H32D	0.9800
C6—H6B	0.9800	C32—H32E	0.9800
C6—H6C	0.9800	C32—H32F	0.9800
C7—C8	1.495 (4)	C33—C34	1.656 (6)
C7—H7A	0.9900	C33—H33A	0.9900
C7—H7B	0.9900	C33—H33B	0.9900
C8—H8A	0.9800	C33'—C34	1.599 (6)
C8—H8B	0.9800	C33'—H33C	0.9900
C8—H8C	0.9800	C33'—H33D	0.9900
N2—C23'^i^	1.508 (4)	C34—H34A	0.9800
N2—C23'	1.508 (4)	C34—H34B	0.9800
N2—C23^i^	1.514 (4)	C34—H34C	0.9800
N2—C23	1.514 (4)	C34—H34D	0.9800
N2—C21'^i^	1.517 (4)	C34—H34E	0.9800
N2—C21'	1.517 (4)	C34—H34F	0.9800
N2—C21	1.533 (4)	C1L—Cl1A	1.765 (3)
N2—C21^i^	1.533 (4)	C1L—Cl1B	1.769 (3)
C21—C22	1.633 (6)	C1L—H1L1	0.9900
C21—H21A	0.9900	C1L—H1L2	0.9900
C21—H21B	0.9900	C2L—Cl2B	1.746 (4)
C21'—C22	1.636 (6)	C2L—Cl2A	1.757 (3)
C21'—H21C	0.9900	C2L—H2L1	0.9900
C21'—H21D	0.9900	C2L—H2L2	0.9900
C22—H22A	0.9800		
			
Cl12—Si1—Cl11	98.50 (4)	C21—C22—H22A	109.5
Cl12—Si1—Si2	113.28 (3)	C21—C22—H22B	109.5
Cl11—Si1—Si2	111.32 (4)	H22A—C22—H22B	109.5
Cl12—Si1—Si5	111.49 (4)	C21—C22—H22C	109.5
Cl11—Si1—Si5	113.90 (4)	H22A—C22—H22C	109.5
Si2—Si1—Si5	108.23 (3)	H22B—C22—H22C	109.5
Cl21—Si2—Cl22	98.77 (4)	C21'—C22—H22D	109.5
Cl21—Si2—Si1	112.27 (4)	C21'—C22—H22E	109.5
Cl22—Si2—Si1	111.72 (3)	H22D—C22—H22E	109.5
Cl21—Si2—Si3	112.69 (3)	C21'—C22—H22F	109.5
Cl22—Si2—Si3	113.01 (3)	H22D—C22—H22F	109.5
Si1—Si2—Si3	108.26 (3)	H22E—C22—H22F	109.5
Cl31—Si3—Cl32	100.07 (3)	N2—C23—C24	110.3 (3)
Cl31—Si3—Si4	111.69 (3)	N2—C23—H23A	109.6
Cl32—Si3—Si4	111.61 (3)	C24—C23—H23A	109.6
Cl31—Si3—Si2	114.12 (3)	N2—C23—H23B	109.6
Cl32—Si3—Si2	111.88 (3)	C24—C23—H23B	109.6
Si4—Si3—Si2	107.47 (3)	H23A—C23—H23B	108.1
Cl41—Si4—Cl42	98.87 (3)	N2—C23'—C24	109.6 (3)
Cl41—Si4—Si3	111.22 (3)	N2—C23'—H23C	109.7
Cl42—Si4—Si3	112.22 (3)	C24—C23'—H23C	109.7
Cl41—Si4—Si5	112.75 (3)	N2—C23'—H23D	109.7
Cl42—Si4—Si5	113.01 (3)	C24—C23'—H23D	109.7
Si3—Si4—Si5	108.60 (3)	H23C—C23'—H23D	108.2
Cl51—Si5—Cl52	99.88 (3)	C23—C24—H24A	109.5
Cl51—Si5—Si1	113.02 (3)	C23—C24—H24B	109.5
Cl52—Si5—Si1	112.15 (3)	H24A—C24—H24B	109.5
Cl51—Si5—Si4	114.27 (3)	C23—C24—H24C	109.5
Cl52—Si5—Si4	110.11 (3)	H24A—C24—H24C	109.5
Si1—Si5—Si4	107.38 (3)	H24B—C24—H24C	109.5
C3—N1—C1	111.90 (19)	C23'—C24—H24D	109.5
C3—N1—C7	108.98 (18)	C23'—C24—H24E	109.5
C1—N1—C7	109.2 (2)	H24D—C24—H24E	109.5
C3—N1—C5	108.79 (19)	C23'—C24—H24F	109.5
C1—N1—C5	108.2 (2)	H24D—C24—H24F	109.5
C7—N1—C5	109.76 (17)	H24E—C24—H24F	109.5
N1—C1—C2	115.0 (2)	C31^ii^—N3—C31	180.0 (4)
N1—C1—H1A	108.5	C31^ii^—N3—C33^ii^	66.3 (3)
C2—C1—H1A	108.5	C31—N3—C33^ii^	113.7 (3)
N1—C1—H1B	108.5	C31^ii^—N3—C33	113.7 (3)
C2—C1—H1B	108.5	C31—N3—C33	66.3 (3)
H1A—C1—H1B	107.5	C33^ii^—N3—C33	180.0
C1—C2—H2A	109.5	C31'^ii^—N3—C31'	180.0 (7)
C1—C2—H2B	109.5	C31'^ii^—N3—C33'^ii^	72.4 (3)
H2A—C2—H2B	109.5	C31'—N3—C33'^ii^	107.6 (3)
C1—C2—H2C	109.5	C31'^ii^—N3—C33'	107.6 (3)
H2A—C2—H2C	109.5	C31'—N3—C33'	72.4 (3)
H2B—C2—H2C	109.5	C33'^ii^—N3—C33'	180.0 (3)
N1—C3—C4	115.5 (2)	N3—C31—C32	110.4 (4)
N1—C3—H3A	108.4	N3—C31—H31A	109.6
C4—C3—H3A	108.4	C32—C31—H31A	109.6
N1—C3—H3B	108.4	N3—C31—H31B	109.6
C4—C3—H3B	108.4	C32—C31—H31B	109.6
H3A—C3—H3B	107.5	H31A—C31—H31B	108.1
C3—C4—H4A	109.5	N3—C31'—C32	110.7 (4)
C3—C4—H4B	109.5	N3—C31'—H31C	109.5
H4A—C4—H4B	109.5	C32—C31'—H31C	109.5
C3—C4—H4C	109.5	N3—C31'—H31D	109.5
H4A—C4—H4C	109.5	C32—C31'—H31D	109.5
H4B—C4—H4C	109.5	H31C—C31'—H31D	108.1
C6—C5—N1	115.2 (2)	C31—C32—H32A	109.5
C6—C5—H5A	108.5	C31—C32—H32B	109.5
N1—C5—H5A	108.5	H32A—C32—H32B	109.5
C6—C5—H5B	108.5	C31—C32—H32C	109.5
N1—C5—H5B	108.5	H32A—C32—H32C	109.5
H5A—C5—H5B	107.5	H32B—C32—H32C	109.5
C5—C6—H6A	109.5	C31'—C32—H32D	109.5
C5—C6—H6B	109.5	C31'—C32—H32E	109.5
H6A—C6—H6B	109.5	H32D—C32—H32E	109.5
C5—C6—H6C	109.5	C31'—C32—H32F	109.5
H6A—C6—H6C	109.5	H32D—C32—H32F	109.5
H6B—C6—H6C	109.5	H32E—C32—H32F	109.5
C8—C7—N1	116.3 (2)	N3—C33—C34	108.3 (3)
C8—C7—H7A	108.2	N3—C33—H33A	110.0
N1—C7—H7A	108.2	C34—C33—H33A	110.0
C8—C7—H7B	108.2	N3—C33—H33B	110.0
N1—C7—H7B	108.2	C34—C33—H33B	110.0
H7A—C7—H7B	107.4	H33A—C33—H33B	108.4
C7—C8—H8A	109.5	N3—C33'—C34	107.8 (3)
C7—C8—H8B	109.5	N3—C33'—H33C	110.1
H8A—C8—H8B	109.5	C34—C33'—H33C	110.1
C7—C8—H8C	109.5	N3—C33'—H33D	110.1
H8A—C8—H8C	109.5	C34—C33'—H33D	110.1
H8B—C8—H8C	109.5	H33C—C33'—H33D	108.5
C23'^i^—N2—C23'	180.0	C33—C34—H34A	109.5
C23^i^—N2—C23	180.0 (3)	C33—C34—H34B	109.5
C23'^i^—N2—C21'^i^	111.7 (2)	H34A—C34—H34B	109.5
C23'—N2—C21'^i^	68.3 (2)	C33—C34—H34C	109.5
C23'^i^—N2—C21'	68.3 (2)	H34A—C34—H34C	109.5
C23'—N2—C21'	111.7 (2)	H34B—C34—H34C	109.5
C21'^i^—N2—C21'	180.0	C33'—C34—H34D	109.5
C23^i^—N2—C21	69.5 (2)	C33'—C34—H34E	109.5
C23—N2—C21	110.5 (2)	H34D—C34—H34E	109.5
C23^i^—N2—C21^i^	110.5 (2)	C33'—C34—H34F	109.5
C23—N2—C21^i^	69.5 (2)	H34D—C34—H34F	109.5
C21—N2—C21^i^	180.0 (4)	H34E—C34—H34F	109.5
N2—C21—C22	107.8 (3)	Cl1A—C1L—Cl1B	110.64 (16)
N2—C21—H21A	110.2	Cl1A—C1L—H1L1	109.5
C22—C21—H21A	110.2	Cl1B—C1L—H1L1	109.5
N2—C21—H21B	110.2	Cl1A—C1L—H1L2	109.5
C22—C21—H21B	110.2	Cl1B—C1L—H1L2	109.5
H21A—C21—H21B	108.5	H1L1—C1L—H1L2	108.1
N2—C21'—C22	108.4 (3)	Cl2B—C2L—Cl2A	111.70 (18)
N2—C21'—H21C	110.0	Cl2B—C2L—H2L1	109.3
C22—C21'—H21C	110.0	Cl2A—C2L—H2L1	109.3
N2—C21'—H21D	110.0	Cl2B—C2L—H2L2	109.3
C22—C21'—H21D	110.0	Cl2A—C2L—H2L2	109.3
H21C—C21'—H21D	108.4	H2L1—C2L—H2L2	107.9
			
C3—N1—C1—C2	52.6 (3)	C23'^i^—N2—C21'—C22	−117.4 (3)
C7—N1—C1—C2	−68.1 (3)	C23'—N2—C21'—C22	62.6 (3)
C5—N1—C1—C2	172.4 (3)	C21—N2—C23—C24	−62.3 (3)
C1—N1—C3—C4	53.9 (3)	C21^i^—N2—C23—C24	117.7 (3)
C7—N1—C3—C4	174.8 (2)	C21'^i^—N2—C23'—C24	−119.9 (3)
C5—N1—C3—C4	−65.6 (3)	C21'—N2—C23'—C24	60.1 (3)
C3—N1—C5—C6	−168.9 (2)	C33^ii^—N3—C31—C32	−49.5 (5)
C1—N1—C5—C6	69.3 (3)	C33—N3—C31—C32	130.5 (5)
C7—N1—C5—C6	−49.8 (3)	C33'^ii^—N3—C31'—C32	57.8 (4)
C3—N1—C7—C8	61.8 (3)	C33'—N3—C31'—C32	−122.2 (4)
C1—N1—C7—C8	−175.7 (2)	C31^ii^—N3—C33—C34	60.0 (5)
C5—N1—C7—C8	−57.2 (3)	C31—N3—C33—C34	−120.0 (5)
C23^i^—N2—C21—C22	118.2 (3)	C31'^ii^—N3—C33'—C34	−58.6 (5)
C23—N2—C21—C22	−61.8 (3)	C31'—N3—C33'—C34	121.4 (5)

Symmetry codes: (i) −*x*, −*y*+1, −*z*+1; (ii) −*x*, −*y*+2, −*z*+1.

### Bis(tetraethylammonium) dichloride decachlorocyclopentasilane dichloromethane disolvate (II) Hydrogen-bond geometry (Å, º)

**Table:**

*D*—H···*A*	*D*—H	H···*A*	*D*···*A*	*D*—H···*A*
C1—H1*B*···Cl42	0.99	2.99	3.829 (3)	144
C2—H2*C*···Cl2	0.98	2.95	3.753 (3)	139
C3—H3*A*···Cl52^iii^	0.99	2.79	3.643 (3)	144
C3—H3*B*···Cl2*B*^iv^	0.99	2.98	3.804 (3)	142
C5—H5*B*···Cl22^v^	0.99	2.89	3.850 (3)	165
C6—H6*B*···Cl21^v^	0.98	2.86	3.630 (3)	136
C7—H7*A*···Cl2	0.99	2.86	3.394 (2)	115
C22—H22*C*···Cl51^vi^	0.98	2.89	3.847 (4)	165
C22—H22*E*···Cl41	0.98	2.90	3.859 (3)	165
C23—H23*B*···Cl1^vi^	0.99	2.98	3.465 (4)	111
C23′—H23*C*···Cl42	0.99	2.87	3.497 (4)	122
C24—H24*C*···Cl41	0.98	2.84	3.793 (3)	164
C24—H24*E*···Cl51^vi^	0.98	2.81	3.771 (3)	165
C24—H24*F*···Cl2*A*^vi^	0.98	2.92	3.778 (3)	147
C31′—H31*C*···Cl31	0.99	2.95	3.434 (5)	111
C32—H32*F*···Cl21^vii^	0.98	2.76	3.584 (4)	142
C33—H33*A*···Cl32^ii^	0.99	2.94	3.515 (4)	118
C33′—H33*D*···Cl41	0.99	2.98	3.630 (5)	124
C34—H34*A*···Cl1*B*^i^	0.98	2.93	3.556 (3)	123
C34—H34*C*···Cl2*B*	0.98	2.89	3.716 (4)	142
C34—H34*A*···Cl1*B*^i^	0.98	2.93	3.556 (3)	123
C1*L*—H1*L*1···Cl12^iii^	0.99	2.90	3.421 (3)	114
C2*L*—H2*L*2···Cl41	0.99	2.96	3.465 (3)	113

Symmetry codes: (i) −*x*, −*y*+1, −*z*+1; (ii) −*x*, −*y*+2, −*z*+1; (iii) −*x*+1, −*y*+1, −*z*; (iv) *x*, *y*, *z*−1; (v) *x*−1, *y*, *z*; (vi) −*x*+1, −*y*+1, −*z*+1; (vii) −*x*+1, −*y*+2, −*z*+1.

## References

[bb1] Dai, X., Anderson, K. J., Schulz, D. L. & Boudjouk, P. (2010). *Dalton Trans.* **39**, 11188–11192. 10.1039/c0dt00925c 20967378

[bb2] Groom, C. R., Bruno, I. J., Lightfoot, M. P. & Ward, S. C. (2016). *Acta Cryst.* B**72**, 171–179. 10.1107/S2052520616003954 PMC4822653 27048719

[bb3] Hengge, E. & Kovar, D. (1977). *J. Organomet. Chem.* **125**, C29–C32.

[bb4] Sheldrick, G. M. (2008). *Acta Cryst.* A**64**, 112–122. 10.1107/S0108767307043930 18156677

[bb5] Sheldrick, G. M. (2015). *Acta Cryst.* C**71**, 3–8.

[bb6] Stoe & Cie (2001). *X-AREA* and *X-RED32*. Stoe & Cie, Darmstadt, Germany.

[bb7] Tillmann, J., Lerner, H.-W. & Bolte, M. (2015). *Acta Cryst.* C**71**, 883–888. 10.1107/S2053229615016484 26422216

[bb8] Tillmann, J., Meyer, L., Schweizer, J. I., Bolte, M., Lerner, H.-W., Wagner, M. & Holthausen, M. C. (2014). *Chem. Eur. J.* **20**, 9234–9239. 10.1002/chem.201402655 24771318

[bb9] Tillmann, J., Meyer-Wegner, F., Nadj, A., Becker-Baldus, J., Sinke, T., Bolte, M., Holthausen, M. C., Wagner, M. & Lerner, H.-W. (2012). *Inorg. Chem.* **51**, 8599–8606. 10.1021/ic301283m 22789129

[bb10] Tillmann, J., Moxter, M., Bolte, M., Lerner, H.-W. & Wagner, M. (2015). *Inorg. Chem.* **54**, 9611–9618. 10.1021/acs.inorgchem.5b01703 26378930

[bb11] Westrip, S. P. (2010). *J. Appl. Cryst.* **43**, 920–925.

