# Supplementary material for: Deca­chloro­cyclo­penta­silanes coordinated by pairs of chloride anions, with different cations, but the same solvent mol­ecules
Source: Acta Crystallogr E Crystallogr Commun. 2017 Nov 21;73(Pt 12):1903–7. doi: 10.1107/S2056989017016310 (PMC5730249; doi:10.1107/S2056989017016310)
Supplement: Supplementary file 4 [file e-73-01903-sup4.pdf]

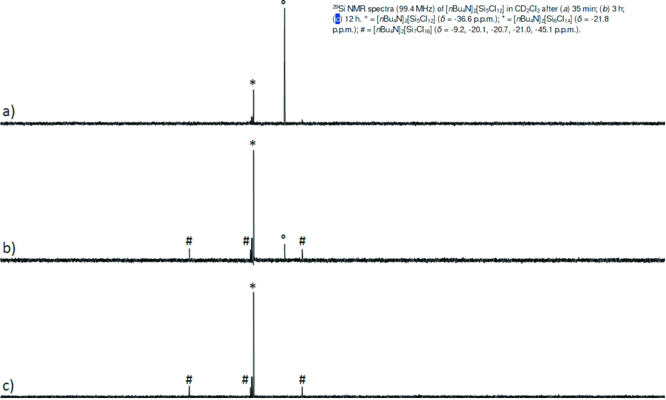

^{29}Si NMR spectra (99.4 MHz) of [nBu$_4$N]$_2$[Si$_5$Cl$_{12}$] in CD$_2$Cl$_2$ after (*a*) 35 min; (*b*) 3 h; (*c*) 12 h. ° = [nBu$_4$N]$_2$[Si$_5$Cl$_{12}$] (δ = -36.6 p.p.m.); * = [nBu$_4$N]$_2$[Si$_6$Cl$_{14}$] (δ = -21.8 p.p.m.); # = [nBu$_4$N]$_2$[Si$_7$Cl$_{16}$] (δ = -9.2, -20.1, -20.7, -21.0, -45.1 p.p.m.).
