# Supplementary figures and images for: Deca­chloro­cyclo­penta­silanes coordinated by pairs of chloride anions, with different cations, but the same solvent mol­ecules
Source: Acta Crystallogr E Crystallogr Commun. 2017 Nov 21;73(Pt 12):1903–7. doi: 10.1107/S2056989017016310 (PMC5730249; doi:10.1107/S2056989017016310)

$^{29}\text{Si}$  CP/MAS NMR spectrum (119.2 Mc) of  $[\text{nBu}_4\text{N}]_2[\text{Si}_2\text{Cl}_{12}]$  ( $\delta = -39.3$  ppm.).

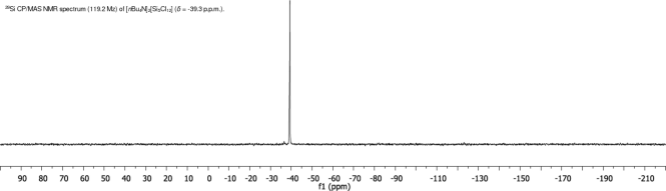

Supplement: Supplementary file 5 [file e-73-01903-sup5.pdf]
